# SID-4/NCK-1 is important for dsRNA import in *Caenorhabditis elegans*

**DOI:** 10.1093/g3journal/jkac252

**Published:** 2022-09-27

**Authors:** Sonya Bhatia, Craig P Hunter

**Affiliations:** Department of Molecular and Cellular Biology, Harvard University, Cambridge MA 02138, USA; Department of Molecular and Cellular Biology, Harvard University, Cambridge MA 02138, USA

**Keywords:** NCK-1, *C. elegans*, RNAi, dsRNA

## Abstract

RNA interference is sequence-specific gene silencing triggered by double-stranded RNA. Systemic RNA interference is where double-stranded RNA, expressed or introduced into 1 cell, is transported to and initiates RNA interference in other cells. Systemic RNA interference is very efficient in *Caenorhabditis elegans* and genetic screens for systemic RNA interference-defective mutants have identified RNA transporters (SID-1, SID-2, and SID-5) and a signaling protein (SID-3). Here, we report that SID-4 is *nck-1*, a *C. elegans* NCK-like adaptor protein. *sid-4* null mutations cause a weak, dose-sensitive, systemic RNA interference defect and can be effectively rescued by SID-4 expression in target tissues only, implying a role in double-stranded RNA import. SID-4 and SID-3 (ACK-1 kinase) homologs interact in mammals and insects, suggesting that they may function in a common signaling pathway; however, a *sid-3; sid-4* double mutants showed additive resistance to RNA interference, suggesting that these proteins likely interact with other signaling pathways as well. A bioinformatic screen coupled to RNA interference sensitivity tests identified 23 additional signaling components with weak RNA interference-defective phenotypes. These observations suggest that environmental conditions may modulate systemic RNA interference efficacy, and indeed, *sid-3* and *sid-4* are required for growth temperature effects on systemic RNA interference silencing efficiency.

## Introduction

RNA interference (RNAi) is sequence-specific gene silencing triggered by natural or experimentally introduced double-stranded RNA (dsRNA) (Fire *et al.* 1998). In many animals, including *Caenorhabditis elegans*, experimentally introduced dsRNA is mobile (Jose and Hunter 2007). This dsRNA mobility allows dsRNA introduced by localized injection and transgenic expression and in some animals by ingestion, to produce a whole-animal systemic silencing response. We previously reported the results of a visual screen for *s*ystemic RNA*i*-*d*efective (Sid) mutants that has led to the identification of dsRNA transport and signal transduction proteins (Winston *et al.* 2002, 2007; Hinas *et al.* 2012; Jose *et al.* 2012).

Characterization of the *sid* genes has so far identified 4 SID proteins. SID-1 is a dsRNA channel protein that selectively transports long dsRNA into cells (Feinberg and Hunter 2003; Shih and Hunter 2011). In *sid-1* mutants, systemic RNAi is undetectable, but expressed or injected dsRNA can cause robust autonomous RNAi (Winston *et al.* 2002). Genetic mosaic and tissue-specific rescue experiments demonstrate that SID-1 is required for import but not export of silencing information (presumably dsRNA) (Winston *et al.* 2002; Jose *et al.* 2009; Whangbo *et al.* 2017). SID-2 is an intestinally expressed transmembrane protein, present at the intestinal lumen, that selectively endocytoses ingested dsRNA (Winston *et al.* 2007; McEwan *et al.* 2012). While SID-2 is required only for feeding RNAi, SID-1 is also required for feeding RNAi; thus, it is presumed that SID-1 releases endocytosed dsRNA into the cytoplasm to initiate RNAi. SID-3 is a broadly expressed ACK1 tyrosine kinase homolog (Jose *et al.* 2012). *sid-3* mutants are partially defective for the import of silencing signals. The requirement of a signal transduction protein suggests that environmental or physiological conditions may regulate systemic RNAi. SID-5 is a small novel protein associated with late endosomes (Hinas *et al.* 2012). Like *sid-3* mutants, *sid-5* mutants are partially defective for RNAi, but *sid-5* is also essential for initiation of parental RNAi (transgenerational silencing) (Wang and Hunter 2017). Here, we report the identification and characterization of *sid-4*.

Several other activities are also required for systemic RNAi. RME-2 is an endocytosis receptor that, in the absence of SID-1, is required to transport dsRNA into oocytes to support parental RNAi (Wang and Hunter 2017). Similar to feeding RNAi, SID-1 activity is subsequently required in the embryo for RNAi silencing, likely to release endosome trapped dsRNA into the cytoplasm to initiate RNAi. In addition, mutations in *rde-10*, *-11*, and *-12* and *rrf-1*, which fail to produce abundant secondary siRNAs resulting in dose-dependent RNAi-silencing defects (Yang *et al.* 2014), are also required for effective systemic RNAi. dsRNA introduced locally (injection or expression) in *rde-12* and *rrf-1* mutants results in RNAi silencing locally, but not systemically, indicating that limiting amounts of dsRNA are transported. Thus, systemic RNAi is likely a dose-sensitive process. Our analysis of *sid-4* provides additional evidence for this hypothesis.

## Materials and methods

### Strains

Unless otherwise indicated, all strains were grown at 20°C on NGM plates seeded with OP50 as a food source (Brenner 1974). Strains used in this analysis are listed in Table 1 and for the candidate SID-3–SID-4-interacting screen in Supplementary Table 1.

**Table 1.** Strains used in this study (see also Supplementary Table 1 for candidate RNAi-defective strains).

Strain	Genotype reference
N2	Wild type
HC57	*ccIs4251 [pSAK2 (myo-3::NGFP-LacZ), pSAK4 (myo-3::mtGFP); dpy-20] I;*
	*qtIs3(myo-2::GFP dsRNA) III;*
	*mIs11[myo-2p::GFP + pes-10p::GFP + F22B7.9::GFP] IV*
HC259	*ccIs4251 I; mIs11 IV; sid-4(qt15)X*
HC119	*ccIs4251 I; mIs11IV; sid-4(qt17)X*
HC158	*ccIs4251 I; qtIs3 III; mIs11 IV; sid-4(qt33)X*
HC160	*ccIs4251 I; qtIs3 III; mIs11 IV; sid-4(qt35)X*
HC114	*ccIs4251 I; qtIs3 III; mIs11 IV; sid-1(qt9)V*
HC122	*ccIs4251 I; sid-2(qt13), qtIs3 III; mIs11 IV;*
HC770	*sid-3(tm342) X*
HC1088	*sid-4(qt33) X*
HC1093	*sid-4(ok694) X [out crossed 8X to N2]*
HC1098	*ccIs4251 I; qtIs3 III; mIs11 IV; sid-4 (ok694) X*
HC1133	*nrIs20 [sur-5::NLS-GFP] IV; sid-4 (ok694) X*
HC1139	*sid-4(ok694) X; qtEx159 [sid-4::GFP]*
HC1140	*nrIs20 IV; sid-4 (ok694) X; qtEx214 [ZK470.5a, PCFJ90(Pmyo-2::mCherry)]; line 1*
HC1141	*nrIs20 IV; sid-4 (ok694) X; qtEx215 [ZK470.5a, PCFJ90(Pmyo-2::mCherry)]; line 2*
HC1142	*nrIs20 IV; sid-4 (ok694) X; qtEx216 [ZK470.5a, PCFJ90(Pmyo-2::mCherry)]; line 3*
HC1143	*nrIs20 IV; sid-4 (ok694) X; qtEx217 [ZK470.5b, PCFJ90(Pmyo-2::mCherry)]; line 1*
HC1144	*nrIs20 IV; sid-4 (ok694) X; qtEx218 [ZK470.5b, PCFJ90(Pmyo-2::mCherry)]; line 2*
HC1145	*nrIs20 IV; sid-4 (ok694) X; qtEx219 [ZK470.5b, PCFJ90(Pmyo-2::mCherry)]; line 3*
HC1146	*sid-4(ok694), sid-3 (tm342) X*
HC1147	*ver-1(ok1738)III; sid-4(ok694)X*
HC1148	*sid-3(tm342), ver-1(ok1738) X*

### Whole genome sequencing

Genomic DNA was extracted from each of the 4 *sid-4* strains: HC259 *sid-4(qt15)*; HC119 *sid-4(qt17)*; HC158 *sid-4(qt33)*; and HC160 *sid-4(qt35)*. Samples were sheared to 250 bp and prepared according to NEB Ultra DNA library kit E7370S. Sample concentrations were quantified via qPCR (Kapa Kit KK4824) and sequenced (Illumina), recovering a total of 250 million reads with 80× coverage. Reads were aligned using bowtie2 and exon variants on linkage group X were called using Samtools. Coding sequence variants in only a single gene, *nck-1*, were identified in all 4 strains.

### SID-4 isoform expression constructs

Full-length *sid-4A* (ZK470.5A) and *sid-4B* (ZK470.5B) cDNA constructs (Open Biosystems) were verified and individually injected (4.5 ng/μl) with a Pmyo-2::mCherry (10 ng/μl) coinjection marker (PCFJ90) and 1 kb DNA ladder (10 ng/μl) (New England Biolabs) into *sur-5::NLS-GFP*; *sid-4(ok694)* (HC1133). For each isoform plasmid, 3 recovered independent complex extrachromosomal array lines (ZK470.5a; HC1140, HC1141, HC1142; ZK470.5b; HC1143, HC1144 HC1145) were analyzed.

### Feeding RNAi assays

L4-young adults were placed on RNAi food (Kamath and Ahringer 2003) at room temperature (unless otherwise specified) and in every case, F_1_ adult progeny were scored for sensitivity to RNAi. Bacteria containing the empty-vector L4440 was used as the control for all feeding RNAi experiments. The following phenotypes were scored for each food: *gfp*, GFP silencing; *dpy-11*, body length (qualitative); *bli-1*, full-body (hyp7) blisters; *unc-45*, paralysis; *unc-22*, twitching; *act-5*, F_1_ larval growth; *fkh-6*, F_1_ fertility (presence of internal eggs); *pos-1*, F_2_ embryonic viability.

### sid-4 mosaic analysis

HC1139 *sid-4(ok694); qtEX159 sid-4::gfp* young adults were cultured on *bli-1* RNAi plates for 3 days. RNAi-sensitive blistered animals were counted and RNAi “resistant” nonblistered animals were collected and scored for GFP expression. All resistant worms expressing detectable GFP were then mounted for widefield microscopy and scored for GFP expression in the hypodermis.

### dsRNA synthesis and injection

*pal-1* dsRNA was made by amplifying a 1.2-kb region from the *pal-1* plasmid (Ahringer library, Kamath and Ahringer 2003) using forward and reverse T7 primers (*pal-1*-F-T7 TAATACGACTCACTATAGGTCCCATTTTAGGCAGTGAGTTA; *pal-1*-R GTTGCCAGCTCGTTATTTTATTG; *pal-1*-F TCCCATTTTAGGCAGTGAGTTTA; *pal-1*-R-T7 TAATACGACTCACTATAGGCTCGAGAAGAAAAAGAACGACAA). A T7-flash Ampliscribe kit was used to make single-stranded sense and anti-sense RNA strands, which were annealed, quantified, diluted as necessary, and injected into either 1 or both gonad arms of *sid-4(ok694)* and N2 animals. Six hours after injection, each recovered animal was singled to an OP50 plate. To score embryonic lethality, the adult was removed after 24 h and laid eggs counted. Three days later, the number of hatched progeny were counted.

### SID-3–SID-4 interactor screen

All known mammalian NCK and ACK physical interactors were obtained from BioGrid (build 3.4.140) and Human protein reference databases (release 9) and all mammalian network information for both was obtained from PathCards (version 4.4) (Keshava Prasad *et al.* 2009; Belinky *et al.* 2015; Chatr-Aryamontri *et al.* 2017). Cross referencing all NCK interactors against both ACK interactors and the network data and ACK interactors against the NCK network data identified 113 unique mammalian proteins. We used wormbase (WS262) to identify 116 *C. elegans* orthologs of these proteins as well as to identify viable mutations. Fifty-three mutant strains were ordered from the *C. elegans* Genetic stock Center (cgc.umn.edu). To test each strain for RNAi defects, young adult hermaphrodites were placed on *fkh-6* RNAi plates and *dpy-11* RNAi plates. Three days later, the *fkh-6* RNAi plates were scored for presence of laid eggs and the *dpy-11* RNAi plates were scored for Dpy progeny.

### Microscopy

Figure 6 images were obtained with a Zeiss Axiovert 200m spinning disk confocal microscope (63× and 100× objectives) equipped with a Hamamatsu Orca-ER digital camera. All the other images were obtained with an Olympus SZX2-TR30PT fluorescent microscope [filter (GFP-470)] equipped with a Hamamatsu digital camera. ImageJ was used to adjust contrast and brightness and Adobe illustrator was used to crop images.

## Results

### *sid-4/nck-1* encodes the *C. elegans* ortholog of the mammalian noncatalytic region of tyrosine kinase adaptor protein

**Fig. 1.**
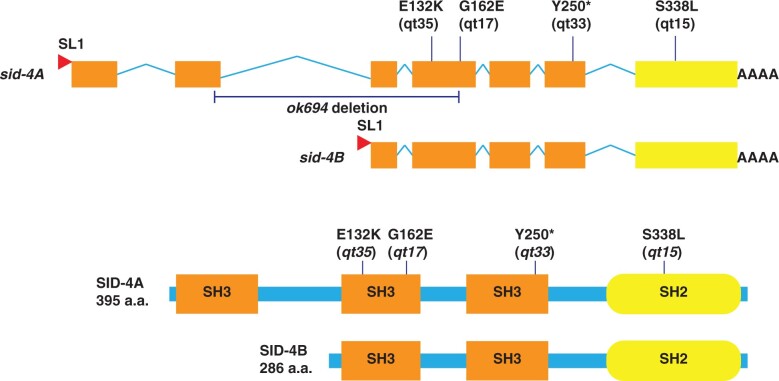
sid-4 encodes NCK-1, a noncatalytic region of tyrosine kinase adaptor protein. Schematic representation of protein-altering SNPs identified in ZK470.5 and their location in SID-4A and SID-4B (adapted from Mohamed and Chin-Sang 2011). *sid-4A* and *sid-4B* are produced by independent promoters and trans-spliced to SL1.

The Sid screen recovered 4 X-linked, noncomplementing alleles that define the *sid-4* locus (Winston *et al.* 2002). Whole genome sequencing of these 4 strains identified protein-altering nucleotide variants in ZK470.5 (*nck-1*) in each isolate: *sid-4(qt15)* [S338L], *sid-4(qt17)* [G162E], *sid-4(qt33)* [Y250-stop], and *sid-4(qt35)* [E132K] (Fig. 1). For subsequent characterization, we used the 1,814 base pair (bp) deletion allele, *sid-4*/*nck-1 (ok694)* (Consortium 2012) (Fig. 1). SID-4/NCK-1 is homologous to adaptor proteins that promote the formation of signaling protein complexes either at the plasma membrane or in the cytoplasm (Buday 1999). Interestingly, SID-3 is homologous to ACK1 (activated CDC42 kinase) (Jose *et al.* 2012) and mammalian and *Drosophila* ACK1 proteins physically interact with NCK proteins (Teo *et al.* 2001; Worby *et al.* 2002). A translational *nck-1 genomic::gfp* reporter is broadly expressed in embryos and adults in a manner similar to SID-3::GFP reporters, supporting the possibility that *sid-3* and *sid-4* may interact to support systemic RNAi (Mohamed and Chin-Sang 2011; Jose *et al.* 2012).

### Both *sid-4* isoforms function to support systemic RNAi

The mammalian NCK homologs, *nck-1/nckα* and *nck-2*/nckβ, may function redundantly as well as have distinct roles in signal transduction (Chen *et al.* 1998; Li *et al.* 2001). *C. elegans sid-4*/*nck-1*, which is equally similar to both mammalian NCK homologs, encodes 2 isoforms: NCK-1A and NCK-1B (Fig. 1). NCK-1A is the larger isoform and contains 3 SH3 domains followed by a single SH2 domain, while NCK-1B lacks the first SH3 domain (Fig. 1). The screen identified missense mutations in the SH2 domain and each of the SH3 domains common to both isoforms (Fig. 1). The lack of a recovered mutation in the first SH3 domain fails to provide the evidence of function for one or the other isoform. The 2 isoforms SID-4A and SID-4B are produced by 2 different *sid-4* promoters and transcription initiation sites (Fig. 1) (Mohamed and Chin-Sang 2011). To test each isoform independently, we rescued the *sid-4(ok694)* with isoform-specific cDNA constructs driven by their endogenous promoters.

**Fig. 2.**
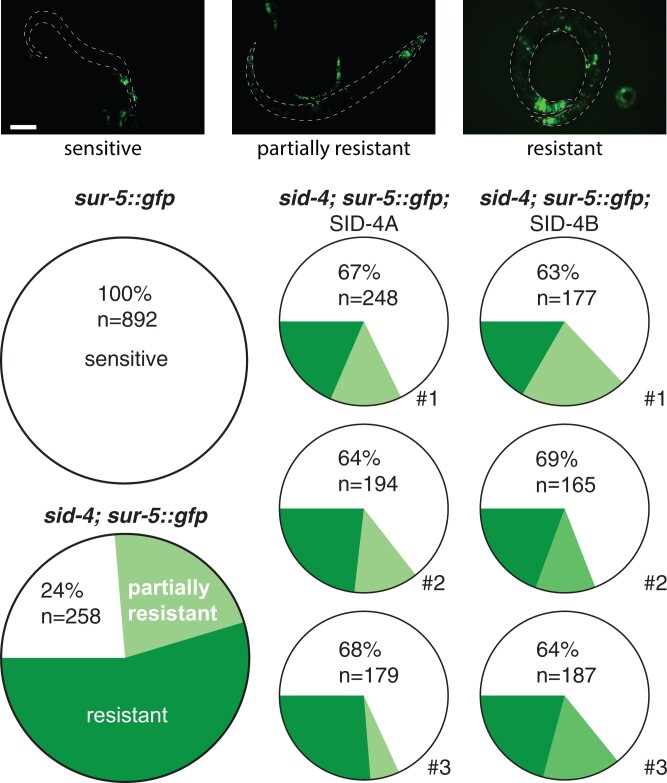
SID-4 isoform rescue of systemic RNAi. Representative images (top) of variable *sur-5*::GFP silencing. Quantification (bottom) of rescue of silencing in 3 independent lines for each SID-4 isoform. Scale bar: 0.1 mm

To score RNAi efficacy in multiple tissues, we monitored the expression of a *sur-5::gfp* transgene that expresses nuclear localized GFP in all somatic cells. The progeny of *sur-5::gfp* hermaphrodites grown on bacteria expressing *gfp* dsRNA are fully silenced, while roughly three-quarters of the progeny of *sid-4(ok694); sur-5::gfp* hermaphrodites grown on bacteria expressing *gfp* dsRNA are resistant or partially resistant to silencing (Fig. 2). We then tested 3 independent SID-4A or SID-4B expressing extrachromosomal array lines for rescue of the silencing defect. GFP expression in nonintestinal cells was blind scored in the progeny of animals placed on *gfp* RNAi food (Fig. 2); the *sur-5::gfp* transgene line is not reliably expressed in the intestine in the presence of other extrachromosomal arrays (Jose *et al.* 2012). We found that both SID-4A and SID-4B similarly and partially rescued silencing in *sid-4; sur-5::gfp* strains (Fig. 2). This result shows that expression of either SID-4 isoform is sufficient to produce SID-4 activity and, therefore, that the first SH3 domain is not required for SID-4 to support systemic RNAi. However, the partial rescue suggest that the isoforms may function together for full activity. To test this, we injected both SID-4A and SID-4B cDNA constructs into the *sid-4(ok694)*; *sur-5::gfp* strain; however, all identified transgenic progeny were developmentally arrested. This phenotype is unlikely to be related to dsRNA transport; thus, we did not investigate further.

### *sid-4* alleles have weak systemic RNAi-defective phenotypes

**Fig. 3.**
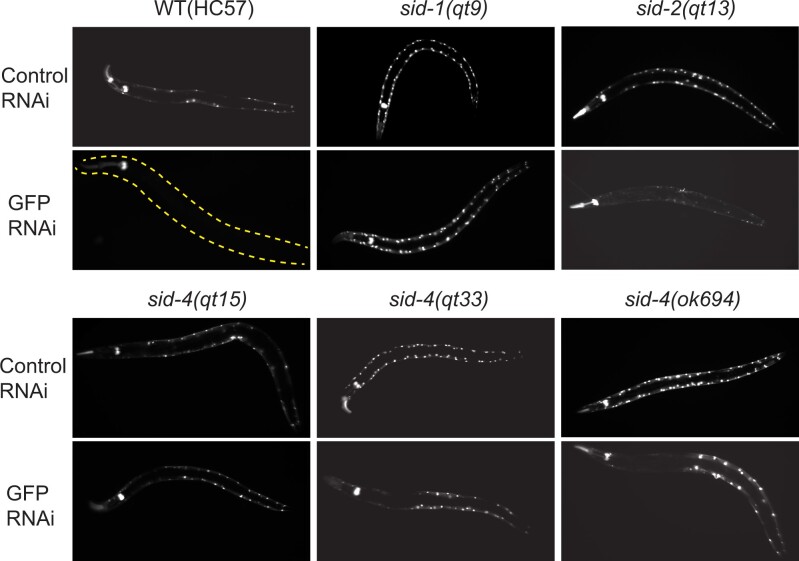
sid-4 mutants are partially defective for RNAi. Adult progeny of indicated *sid* mutant L4 hermaphrodites in the HC57 background (pharyngeal GFP; body wall muscle GFP; pharynx expressed *gfp* dsRNA) placed on bacteria expressing *gfp* dsRNA (GFP RNAi) or L440 empty-vector bacteria (control RNAi). All animals approximately 1 mm. *sid-4 (ok694)* and *sid-4 (qt33)* progeny showed indistinguishable penetrance and expressivity of systemic RNAi-silencing defects (Supplementary Fig. 1).

The above analysis showed that *sid-4* mutants partially disable RNAi silencing. To directly compare *sid-4* systemic silencing defects to that of other Sid mutants, we crossed the deletion allele *sid-4(ok694)* into the transgenic HC57 background used for the screen. In this transgenic strain, pharyngeal expressed *gfp* dsRNA autonomously silences pharyngeal GFP and systemically silences GFP in anterior body wall muscle cells. When cultured on *gfp* dsRNA expressing bacteria, the ingested dsRNA silences GFP in all body wall muscle cells. A *sid-1* mutant in this transgenic background disrupts the silencing of the body wall muscle GFP from both pharyngeal expressed *gfp* dsRNA and ingested bacterial *gfp* dsRNA (Fig. 3). In contrast, *sid-2* mutants disrupt only silencing in response to ingested dsRNA. Thus, *sid-2* mutants in the transgenic background have the same silencing phenotype whether grown on normal bacteria or bacteria expressing *gfp* dsRNA (Fig. 3). In contrast to *sid-1* and *sid-2* mutants, *sid-4(qt33)* and *sid-4(ok694)* show incomplete systemic RNAi defects (Fig. 2a and Supplementary Fig. 1). Like *sid-1* and *sid-2*, neither allele disrupts pharyngeal silencing, confirming that the mutations do not noticeably disrupt RNAi. However, in contrast to *sid-1* and *sid-2*, many anterior body wall muscle cells are silenced when grown on bacteria expressing *gfp* dsRNA. While this indicates that *sid-4* is not required for the uptake of ingested dsRNA, the lack of complete silencing may indicate a dose-dependent response to exported pharyngeal expressed or ingested *gfp* dsRNA. Detailed comparison of the extent of body-wall muscle GFP silencing shows no discernable difference between the 2 *sid-4* alleles (Supplementary Fig. 1).

**Fig. 4.**
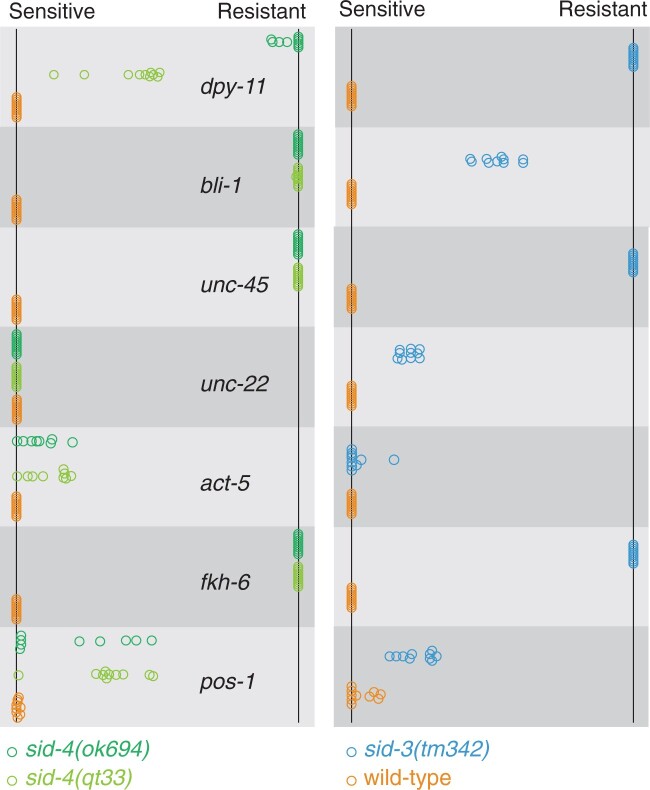
sid-4 and *sid-3* have similar RNAi phenotypes. The adult progeny (F_1_) of L4 animals (F_0_) placed on RNAi foods were scored for RNAi sensitivity (fraction sensitive on each plate). Each circle represents the plate mean sensitivity for each F_0_ (*n* = 10) with ≥150 F_1_’s for each F_0_. 100% sensitive (left) to 100% resistant (right).

Weak *sid-1* alleles show gene-specific patterns of silencing that correlate with strong and weak RNAi foods (Whangbo *et al.* 2017). We tested both *sid-4* alleles on that same set of strong and weak RNAi foods. Wild-type worms were completely sensitive (100% silencing) to all these RNAi foods (Fig. 4), while strong *sid-1* and *sid-2* mutant worms were completely resistant (0% silencing, not shown). *sid-4(qt33)* and *sid-4(ok694)* worms were completely or nearly completely resistant to *fkh-6* (gonad), *unc-45* (muscle), and *bli-1* (hypodermis); completely or mostly sensitive to *act-5* (intestine) and *unc-22* (muscle); and partially resistant to *pos-1* (germline) and *dpy-11* (hypodermis). The discrepancy in sensitivity to *dpy-11* between *qt33* (nonsense) and *ok694* (deletion) indicates that the nonsense mutant *qt33* may have residual activity, perhaps reflecting tissue-specific read-through translation of the stop codon (Fig. 4). The similarity in the pattern of RNAi sensitivity and resistance between strong *sid-4* alleles and weak *sid-1* alleles is consistent with *sid-4* mutations compromising systemic RNAi generally, rather than reflecting a tissue or dsRNA delivery-specific effect.

Because *sid-3* is also a weak Sid mutant and mammalian and *Drosophila* SID-3 and SID-4 homologs have been shown to interact, we tested *sid-3(tm342)* on the same panel of RNAi foods (Fig. 4). We observed a similar, but not identical pattern of resistance and sensitivity. These results are consistent with *sid-3* and *sid-4* acting in concert to promote systemic RNAi. In summary, these results indicate that the *sid-4* null phenotype is not a tissue-specific defect but an incomplete RNAi defect.

### *sid-4* is a dose-dependent Sid mutant

*sid-4* mutant worms retain RNAi-silencing activity in the pharynx and anterior body wall muscle cells (Fig. 2a) indicating that *sid-4* mutations do not compromise RNAi-silencing activity. However, weak RNAi-defective (Rde) mutants that are suppressed by high levels of pharyngeal expressed dsRNA can produce wild-type silencing in the pharynx (Yang *et al.* 2014). To explicitly test *sid-4* mutants for a weak Rde phenotype, we injected *pal-1* dsRNA directly into the syncytial germline of wild-type and *sid-4* mutant hermaphrodites and scored the frequency of the *pal-1*(RNAi) phenotype, embryonic lethality. Because the germline is syncytial, injections into the anterior or posterior gonad do not require dsRNA transport for effective silencing. If *sid-4* mutants are weak RNAi defective, then wild-type and *sid-4* mutant worms should show distinct embryonic lethality frequencies in response to decreasing injected dsRNA dose. We injected both gonad arms to eliminate the effect of gonad to gonad transfer.

**Fig. 5.**
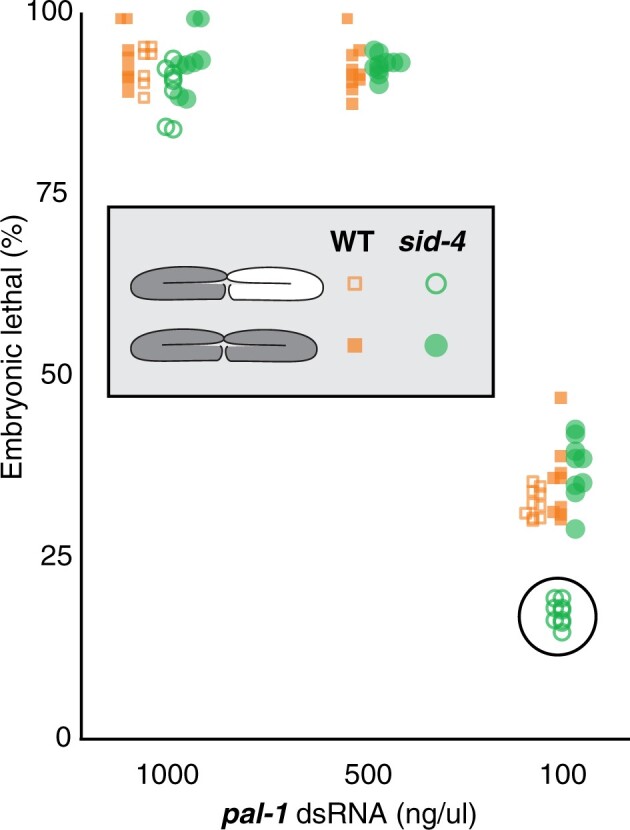
sid-4 is a dose-dependent Sid. Wild-type and *sid-4(ok694)* young adults were injected with varying *pal-1* dsRNA concentrations into 1 (open symbols) or both gonad arms (filled symbols) and then 6 h postinjection singled and allowed to lay eggs for 24 h. The fraction of hatched eggs for each injected adult (*n* ≥ 10) was scored 3 days later.

Furthermore, for each dsRNA concentration, we used a single needle and identical injection times to limit variability and enable direct comparison between strains. We found that both wild-type and *sid-4(ok694)* mutants showed proportionate changes in embryonic lethality when injected with changing concentrations of *pal-1* dsRNA (Fig. 5). This indicates that *sid-4* mutants have a wild-type level of RNAi activity in the germline and are not weak Rde mutants.

We used the same assay to determine whether *sid-4* is required to transfer silencing information between gonad arms. In wild-type animals, injecting a single gonad arm can result in 100% embryonic lethality while in strong *sid-1* mutants only progeny from the injected gonad are affected (Winston *et al.* 2002; Wang and Hunter 2017). To test *sid-4(ok694)* mutants for systemic RNAi defects (Sid), we injected single gonad arms with either a high or low *pal-1* dsRNA dose. Consistent with effective systemic RNAi, there was no detectable difference in embryonic lethality among the progeny of wild-type worms injected with *pal-1* dsRNA into either a single or both gonad arms at either high or low dsRNA dose (Fig. 5). Similarly, the frequency of embryonic lethality among the progeny of *sid-4(ok694)* animals injected with the high concentration of *pal-1* dsRNA into either a single or both gonad arms was nearly identical (Fig. 5). However, the frequency of embryonic lethality among the progeny of *sid-4(ok694)* animals injected with the low concentration of *pal-1* dsRNA into single gonad arm was half that of animals injected into both gonad arms (Fig. 5). This dose-dependent systemic RNAi result is consistent with inefficient transport of dsRNA between gonad arms, indicating that *sid-4* is a weak Sid mutant.

### *sid-4* is import defective

**Fig. 6.**
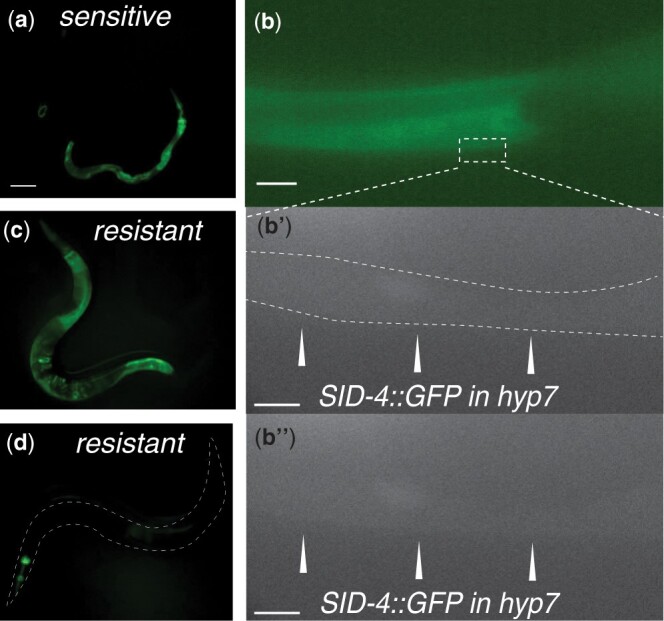
sid-4 is required in the importing tissue. Progeny of *sid-4(ok694); [Ex: sid-4*::gfp] animals were scored for *bli-1* RNAi sensitivity and SID-4::GFP expression. a) A SID-4::GFP positive *bli-1* RNAi-sensitive animal. Scale bar: 0.1 mm. b, b′, and b″) Confocal image of a *bli-1* RNAi-sensitive animal showing SID-4 GFP expression in hyp7. In (b), the bright gut GFP obscures dim diffuse GFP in syncytial Hyp7, which (b′) is outlined in the enlarged gray-scale image that excludes the bright intestinal signal. b″) The same image without outlining. Scale bars: 25 and 5 µm. c) Intestine and d) pharyngeal SID-4::GFP expression in *bli-1* RNAi-resistant animals.

We used genetic mosaic analysis to determine whether *sid-4* is required in importing cells for efficient feeding RNAi. Specifically, we rescued *sid-4(ok694)* animals with a *sid-4::gfp* construct maintained as a mitotically unstable extrachromosomal array. About 82% of the progeny inherited the array and were GFP positive and formed full-body blisters when cultured on *bli-1* RNAi food. The remaining 18% of the progeny did not inherit the array and were mostly GFP negative and fully resistant to *bli-1* RNAi. *bli-1* is expressed in the large syncytial cell hyp7 (139 nuclei, 29 from embryonic cells that fuse to form hyp7 and 110 from postembryonic cells that fuse with hyp7), which produces the full-body blister in *bli-1* RNAi-treated animals (Chisholm and Hsiao 2012). Among 976-resistant worms, we identified 74 with detectable GFP expression. If *sid-4* is required for import, then in these resistant animals, we would expect all or most of the hyp7 nuclei to lack the *sid-4::gfp* array and fail to express detectable GFP in hyp7. Indeed, in none of these animals, did we detect GFP expression in hyp7. It is notable that all of the hyp7 nuclei arise from descendants of 3 distinct early embryonic cells (ABarp, ABp, and C; Supplementary Fig. 2); thus, to produce a *sid-4::gfp* mosaic lacking *sid-4::GFP* expression in most hyp7 nuclei would require 2 or 3 losses. For example (see Supplementary Fig. 1b), the array may fail to segregate to AB at the first division and fail to segregate to P2 at the second division producing a mosaic animal that lacks *sid-4::gfp* in all hyp7 nuclei but expresses *sid-4::gfp* in the posterior pharynx, the gut, and anterior body wall muscle cells. Twenty-two *bli-1* RNAi-resistant animals were identified that had pharynx and gut GFP expression and lacked detectable GFP in hyp7 (Fig. 6c). As a second example (Supplementary Fig. 1c), the array could be lost in P1, ABp, and ABar(pp), producing an animal that lacks *sid-4::gfp* in all hyp7 nuclei, but that expresses GFP in the anterior pharynx only. Fifty-two animals were recovered that are consistent with this scenario (Fig. 6d). It is important to note that by selecting for resistant animals that maintained some GFP, we greatly enriched for mosaic animals that failed to segregate the array at multiple cell divisions. In summary, the results of the *sid-4* genetic mosaic analysis indicate that *sid-4* is required in the target cell for effective RNAi.

### *sid-3* and *sid-4* genetically interact but do not function in a simple linear pathway

**Fig. 7.**
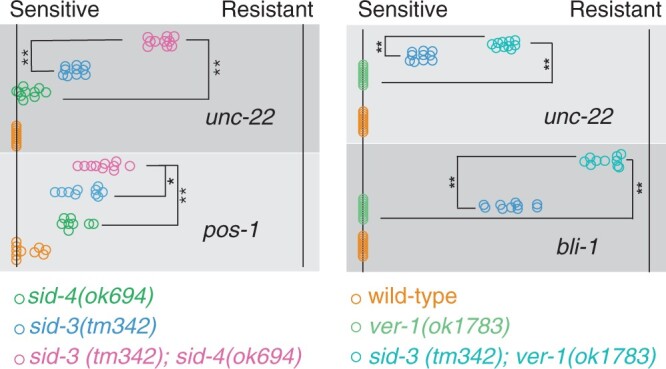
Double Sid weak mutants show enhanced RNAi resistance. The F_1_ adult progeny of F_0_ L4 animals placed on RNAi foods were scored for RNAi sensitivity (fraction sensitive on each plate). Each circle represents the plate mean sensitivity for each F_0_ (*n* = 10) with ≥40 F_1_’s per F_0_. 100% sensitive (left) to 100% resistant (right). *t*-test *P*-values: *<0.01, **<0.0005.

Mammalian and *Drosophila* SID-3/ACK1 and SID-4/NCK-1 homologs interact (Teo *et al.* 2001; Worby *et al.* 2002); thus, it is likely that they do so in *C. elegans* as well. Both *sid-4/nck-1* and *sid-3* fluorescent protein fusion constructs are broadly expressed cytoplasmic proteins that localize to intracellular membranes (Mohamed and Chin-Sang 2011; Jose *et al.* 2012). Furthermore, both *sid-3* and *sid-4* are weak Sid mutants required in importing cells for effective systemic RNAi (Jose *et al.* 2012; Figs. 3–6). If these genes function together as a kinase and exclusive adapter, then we expect the double mutant to resemble both single mutants. To test this, we constructed and tested a *sid-3(tm342); sid-4(ok694)* double mutant on a broad panel of RNAi foods (Supplementary Fig. 3). For many of the foods, the sensitivity of either single mutant was maximal or minimal, precluding interpretation of the double mutant sensitivity. But for 2 foods, *unc-22* (muscle) and *pos-1* (germline), the sensitivity of both single mutants was intermediate, and in both cases, the double mutant was less sensitive (Fig. 7). This result shows that the effect of *sid-3* and *sid-4* are at least partially additive, indicating that in these 2 tissues, these 2 signaling proteins do not function in a simple linear pathway but likely interact independently with other signaling proteins to modulate systemic RNAi silencing.

### *C. elegans* homologs of mammalian NCK/ACK-interacting proteins have weak RNAi phenotypes

The above double mutant analysis suggests that SID-3/4-interacting proteins may modulate systemic RNAi. To identify candidate *C. elegans sid-3* and *sid-4*-interacting proteins, we compiled a list 113 mammalian proteins that likely interact (directly or indirectly) with both an NCK and an ACK ortholog (see *Materials and Methods*). Among 116 *C. elegans* orthologs of these proteins, 80 viable mutants have been described and we tested 52 by feeding RNAi for reduced sensitivity to *fkh-6* and *dpy-11* RNAi food (Supplementary Table 1). Twenty-two candidates were detectably resistant to *fkh-6* RNAi, 7 of which were also partially resistant to *dpy-11* RNAi, and 1 strain was only partially resistant to *dpy-11* RNAi (Table 2). In addition, 5 of the 29 strains that were fully sensitive produced enhanced Dpy-11 phenotypes, possibly signifying enhanced RNAi.

**Table 2.** RNAi-defective SID-3–SID-4 candidate-interacting proteins.

Strain	Genotype^a^	*fkh-6* (RNAi) eggs on plate?	*dpy-11* (RNAi) % non-Dpy adults	Homology	Lethal null?
		(*n* = 2)	(*n* = 2)		
N2	WT	None	0	–	–
CB96	*vab-2(e96) IV*	Many	44	Ephrin ligand	Y
CZ375	*vab-1(e856) II*	Many	0	Ephrin receptor	Y
EM305	*efn-4(bx80) IV*	Many	15	Ephrin ligand	Y
RB942	*cdc-42(ok825) II*	Many/few	19	GTPase	Y
MT12615	*mys-1(n3681) V*	Many	0	Histone acetylase	Y
VC1263	*ver-1(ok1738) III*	Many	0	Receptor tyrosine kinase	
RB2513	*C26C6.6(ok3481) I*	Many	29	Lim domain	
VC664	*ras-1(ok977) II*	Few	18	GTPase	
MT4434	*ced-5(n1812) IV*	Few	15	DOCK	
CB3257	*ced-2(e1752) IV*	Few	0	SH2/SH3 adapter	
CX51	*dyn-1(ky51) X*	Few	0	Dynactin	Y
MT5267	*soc-1(n1789) V*	Few	0	Pleckstrin homology domain	
RB1591	*ddr-1(ok1956) X*	Few	0	Tyr kinase	
RB689	*pak-1(ok488) X*	Few	0	Ser/Thr kinase	Y
NW1549	*efn-2(ev658) IV; efn-3(ev696) X*	Few	0 (*n* = 1)	Ephrin ligand	Sterile
CZ414	*vab-1(e699) II*	Few	0	Ephrin receptor	Y
RB1267	*ensh-1 (ok1349) X*	Few	0	Fibrogenin like	
RB1751	*rga-5(ok2241) IV*	Few	0	RhoGAP	
RB759	*akt-1(ok525) V*	Few	0	Ser/Thr kinase	
ZD500	*hecw-1(ok1347) III*	Few	0	E3 ubiquitin ligase	
RB776	*kin-32(ok166) I*	Few	0	Tyr kinase	

a Some strains include nonrelevant mutations, listed in full in Supplementary Table 1.

Among the top candidates was the receptor tyrosine kinase VER-1 (Table 2), which is orthologous to the mammalian VEGF Receptor 1, an interactor of NCK1 (Igarashi *et al*. 1998). In *C. elegans*, VER-1 is expressed in the intestine and neuronal sheath cells (Procko *et al*. 2012). To explore the RNAi sensitivity of the *ver-1(ok1783)* deletion mutant more thoroughly, we scored the RNAi phenotypes of the progeny of adult animals placed on a panel of RNAi foods targeting a variety of tissues (Supplementary Fig. 4). The progeny of wild-type animals grown on *fkh-6* RNAi food fail to lay any eggs and while *ver-1(ok1783)* animals lay eggs (Table 2); however, this analysis showed that the proportion of egg-laying adults is small (Supplementary Fig. 3). Similarly, the penetrance of *dpy-11* RNAi resistance was low. These results suggest that *ver-1* is a very weak RNAi-defective mutant and would most likely not be recovered in a forward genetic screen. Because *sid-3, sid-4* double mutants show a stronger RNAi resistance phenotype than either single mutant, we constructed and tested *ver-1(ok1783); sid-3(tm342)* double mutant on the same panel of RNAi foods (Supplementary Fig. 3). Consistent with VER-1 acting in the SID-4 pathway, we found that the double mutant was more resistant to feeding RNAi targeting *unc-22* and *bli-1* than either single mutant (Fig. 7).

In summary, 23 of 52 candidate SID-3/4-interacting protein mutants had detectable RNAi defects, supporting the hypothesis that additional signaling components and likely pathways modulate systemic RNAi. Null mutants for many of these genes are lethal, thus perhaps explaining the low penetrance and expressivity of the observed RNAi defects. This, combined with the high level of noise in the experimental assays, makes it challenging to verify the significance of these interactions.

### SID-3/SID-4 is required for temperature-dependent RNAi silencing

**Fig. 8.**
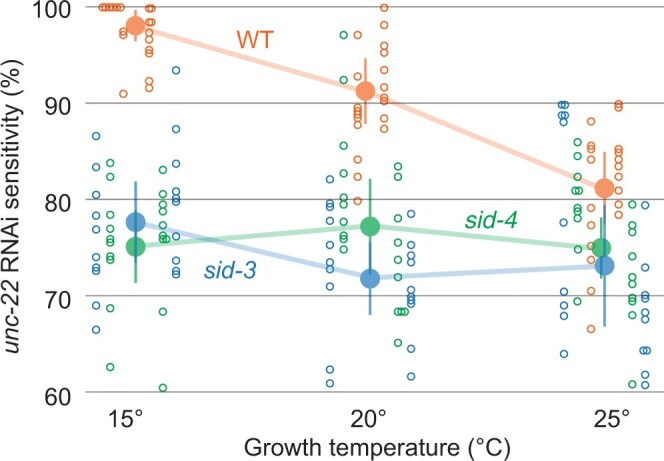
sid-3 and *sid-3* temperature-dependent systemic RNAi. F_1_ progeny of F_0_ animals placed on *unc-22* food at the indicated temperature were scored in 3 mM Levamisole for paralysis/twitching (sensitivity). Mean (large filled circle), 95% confidence interval (vertical line), and each F_0_ plate mean (small unfilled circle) are shown for each genotype temperature. Distribution of 10 plate means for 2 independent trials is shown to the left and right of each mean.

Efficient systemic RNAi requires signaling proteins, indicating that physiological or environmental conditions may modulate systemic RNAi. We previously reported that temperature affects systemic RNAi; we noted more efficient silencing of body wall muscle GFP in response to exported pharyngeal *gfp* dsRNA at 20°C than at 25°C (Winston *et al.* 2002). To extend this analysis, we determined whether temperature can affect the silencing of the endogenous gene *unc-22* in response to ingested *unc-22* dsRNA. The progeny of 10 singled wild-type L4’s placed on *unc-22* RNAi food at 15, 20, and 25°C were scored for *unc-22* twitching as adults. Consistent with the previous findings, 2 independent trials showed more efficient silencing at 15°C and less efficient silencing at 25°C (Fig. 8). Thus, the temperature effect on systemic RNAi silencing is independent of the method of dsRNA delivery (expression vs. ingestion) and target gene (transgene vs. endogenous). We then repeated the experiment with *sid-3* and *sid-4* mutants (Fig. 8). We found that the progeny of both *sid-3* and *sid-4* worms showed less efficient silencing at all 3 growth temperatures. This experiment was then repeated in a second large trial (Fig. 8), which confirmed that *sid-3* and *sid-4* mutants are indistinguishable from each other and wild type at 25°C and that the penetrance of both mutants were most different from wild type at 15°C (*P* < 10^−8^; *t*-test). These results indicate that *sid-3* and *sid-4* do not contribute to systemic RNAi at 25°C and are required primarily for the low temperature enhanced penetrance of systemic RNAi.

## Discussion

The identity of SID-4 as a NCK homolog complements the identity of SID-3 as an ACK1 homolog, one of the presumed SID-4/NCK-1-binding partners. Establishing that these proteins interact to regulate systemic RNAi in *C. elegans* and understanding what conditions regulate their activity will lead to further insight into the mechanisms and functions of systemic RNAi. Unlike the dsRNA transporters SID-1 and SID-2, these 2 proteins have been implicated in a variety of presumably systemic RNAi-independent functions in *C. elegans*, including cell migration (Martynovsky *et al.* 2012; Mohamed *et al.* 2012), virus entry (Jiang *et al.* 2017; Tanguy *et al.* 2017), and endocytic trafficking (Lazetic *et al.* 2018) and cell polarity (Goh *et al.* 2012). All these functions impinge on endocytosis.

Endocytosis has been implicated in feeding RNAi as well as SID-1-independent parental RNAi. In parental RNAi, the endocytosis receptor RME-2 is required for SID-1-independent transfer of extracellular dsRNA into oocytes (Wang and Hunter 2017). SID-1 is then subsequently required in the embryo for RNAi silencing, presumably to release the endocytosed dsRNA into the cytoplasm to initiate RNAi. In feeding RNAi, the SID-2 transmembrane protein, which is expressed in the intestine and localizes to the apical (lumenal) membrane, appears to act as a dsRNA specific endocytosis receptor (Winston *et al.* 2007; McEwan *et al.* 2012). SID-1 is also required for feeding RNAi induced silencing in intestinal cells, again, presumably to release endocytosed dsRNA into the cytoplasm to initiate RNAi. Furthermore, homologs of several of the candidate SID-3/SID-4-interacting proteins that had detectable Rde phenotypes have roles in or are implicated in endocytosis, including *dyn-1* (dynactin), *pak-1*(p21 activated kinase), and *cdc-42*. It should be emphasized that only viable alleles of candidate SID-3–SID-4-interacting proteins were tested and for many of these genes null alleles are inviable (Table 2). Thus, these weak RNAi-defective phenotypes are associated with, in many cases, weak loss-of-function alleles of essential genes. Thus, even if endocytosis is critical for dsRNA transport, we are unlikely to recover strong viable Sid mutants disrupted for endocytosis.

The RNAi potency that makes systemic RNAi possible also makes it difficult to identify and analyze weak RNAi-defective mutants. The vanishing small amounts of dsRNA that are transported into recipient cells are processed and then amplified by RNA-directed RNA polymerases to produce abundant secondary siRNAs. Thus, to observe a Sid phenotype, dsRNA transport must be nearly blocked, not merely disrupted. This is aptly illustrated by the dose sensitivity we documented for *sid-4* (Fig. 5). This dose sensitivity may also explain our results with *fkh-6(RNAi)*. Among the tested RNAi foods, *sid-4* mutants were most resistant to *fkh-6* silencing (Fig. 4). A reasonable interpretation for this high penetrance and expressivity is that *fkh-6* is a poor RNAi target, such that even a modest reduction in dsRNA transport is sufficient to cause a silencing defect. These observations indicate that it will be difficult to directly observe disrupted transport of dsRNA in *sid-4* mutants.

The original large visual screen identified many alleles in *sid-1* and *sid-2* with a strong Sid phenotype and relatively few alleles in *sid-3*, *sid-4*, and *sid-5*, which produce a weak Sid phenotype. This bias likely reflects the relative ease of identifying worms with bright GFP in many cells in the strong mutants (*sid-1* and *sid-2* mutants) vs. the variable and relatively sparse GFP expression in the weak mutants (*sid-3*, *sid-4*, and *sid-5*, as well as a partial loss-of-function *sid-1* alleles; Whangbo *et al.* 2017). Our screen and analysis of candidate SID-3/4-interacting proteins extends this pattern, suggesting that most or all strong Sid mutants have been identified. Our analyses also showed that *sid-3* and *sid-4* are dose-dependent SIDs, that is, increasing the dose of dsRNA bypasses the dsRNA import defect.

Furthermore, and unexpectedly, we discovered that combining 2 weak mutants resulted in a stronger Sid phenotype, suggesting that multiple independent processes contribute to dsRNA import. Some of these processes may involve putative NCK/ACK1-interacting proteins, several of which have weak RNAi-defective phenotypes. Finally, the *sid-3*- and *sid-4*-dependent process is most apparent at lower growth temperatures, confirming that the temperature effect on silencing is due to more efficient systemic RNAi at lower growth temperature.

## Supplementary Material

jkac252_Supplemental_Figures

jkac252_SupplementalTable_S1

## Data Availability

Strains not available at the CGC (cgc.umn.edu) are available upon request. The authors affirm that all data necessary for confirming the conclusions of the article are present within the article, figures, and tables. Supplemental material is available at G3 online.
