# Supplementary material for: SID-4/NCK-1 is important for dsRNA import in Caenorhabditis elegans
Source: G3 (Bethesda). 2022 Sep 27;12(11):jkac252. doi: 10.1093/g3journal/jkac252 (PMC9635667; doi:10.1093/g3journal/jkac252)
Supplement: jkac252_Supplemental_Figures [file jkac252_supplemental_figures.pdf]

Supplemental Figures

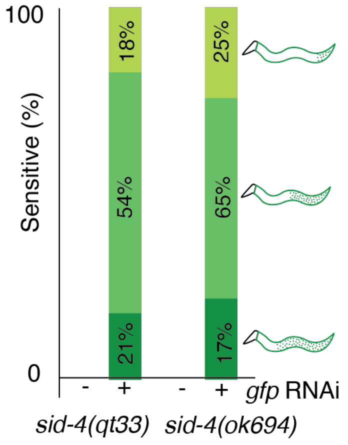

Figure S1. Expressivity and penetrance of *sid-4* alleles.
sid-4 (ok694) and *sid-4 (qt33)* progeny showed indistinguishable penetrance and expressivity of RNAi silencing defects on bacteria expressing *gfp* dsRNA (n≥400). The examples shown in Figure 3 show the most abundant expressivity class (Medium green). HC57 showed 100% sensitivity and *sid-1(qt9)* showed 0% sensitivity (not shown).

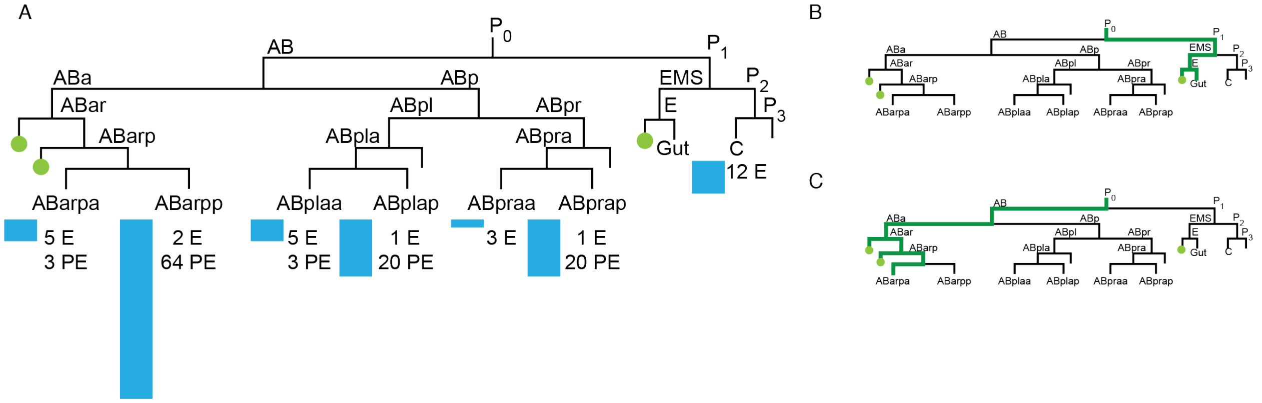

Figure S2. Lineal relatedness of cells that fuse to form syncytial hypodermal cell hyp7.

A) Lineage tree showing the cell divisions (vertical lines) leading to the seven early blastomeres that give rise to embryonic (E) or postembryonic (PE) cells that fuse with hyp7. The blue bar show the relative contributions to hyp7 and the green circles show the origins of pharyngeal muscle cells that express *sid-4::gfp*.

B, C) Examples of extrachromosomal array transmission patterns that could generate the patterns observed in *bli-1* (RNAi) resistant *sid-4::gfp* positive mosaic animals (Figure 6).

B) At the first division the array segregates to P1 but not to AB and at the second division segregates to EMS but not to P2. The resulting animal expresses *sid-4::gfp* in the intestine and cells of the posterior pharynx, but lacks *sid-4::gfp* in all hyp7 nuclei.

C) At the first division the array segregates to AB but not to P1, at the second division segregates to ABa but not to ABp, and at a subsequent division segregates to ABar, ABarp, or ABarpp. The resulting animal expresses *sid-4::gfp* in cells of the anterior pharynx, but lacks *sid-4::gfp* expression in all or most hpy7 nuclei.

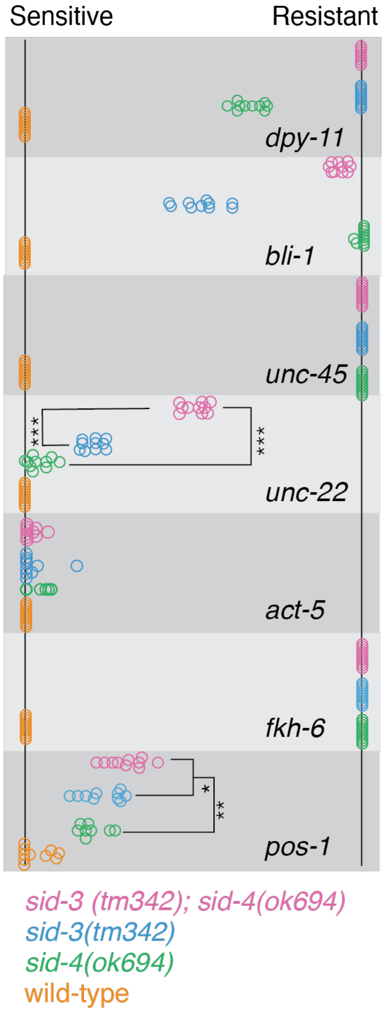

Figure S3 (supporting Figure 7A) RNAi sensitivity of *sid-3* and *sid-4 single* and *sid-3; sid-4* double mutants on a panel of RNAi foods. The F_1 adult progeny of F_0 L4 animals placed on RNAi foods were scored for RNAi sensitivity (fraction sensitive on each plate). Each circle represents the plate mean sensitivity for each F_0 (n=10) with $\geq$40 F_1's per F_0. P-values (t-test). * <0.01, ** <0.0005, *** <2.3×10^{-15}.

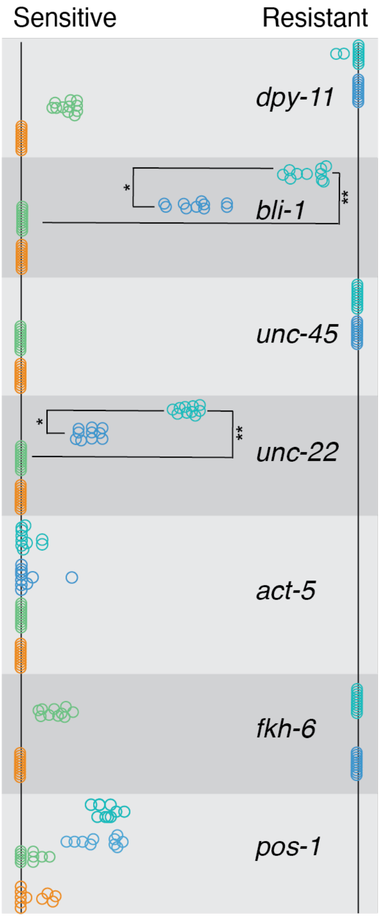

Figure S4 (supporting Figure 7B) RNAi sensitivity of *sid-3* and *ver-1 single* and *sid-3; ver-1* double mutants on a panel of RNAi foods. The F_1 adult progeny of F_0 L4 animals placed on RNAi foods were scored for RNAi sensitivity (fraction sensitive on each plate). For *fkh-6*, the proportion of F1 adults that contained embryos was scored. Each circle represents the plate mean sensitivity for each F_0 (n=10) with $\geq$40 F_1's per F_0. P-values (t-test). * <2.7x10^{-11}, ** <1.6x10^{-21}.
