# Supplementary material for: SID-4/NCK-1 is important for dsRNA import in Caenorhabditis elegans
Source: G3 (Bethesda). 2022 Sep 27;12(11):jkac252. doi: 10.1093/g3journal/jkac252 (PMC9635667; doi:10.1093/g3journal/jkac252)
Supplement: jkac252_SupplementalTable_S1 [file jkac252_supplementaltable_s1.docx]

| Table S1 |  |  |  |  |
| --- | --- | --- | --- | --- |
| Strain | *genotype* | *fkh-6* (RNAi)  eggs on plate (n=2) | *dpy-11* (RNAi)  average % non-Dpy adults (n=2) | notes |
| CB96 | *vab-2(e96) IV* | many | 44 | Ephrin Ligand |
| RB2513 | *C26C6.6(ok3481) I* | many | 29 | Lim domain |
| EM305 | *efn-4(bx80) IV;  him-5(e1490) V* | many | 15 | Ephrin Ligand |
| RB942 | *cdc-42(ok825) II* | many/few | 19 | GTPase |
| VC610 | *ver-3(ok891) X* | many/few | 11 | Receptor Tyrosine Kinase |
| MT12615 | *mys-1(n3681) V* | many (n=1) | 0 | Histone Acetylase |
| CZ375 | *vab-1(e856) II* | many | 0 | Ephrin Receptor |
| VC1263 | *ver-1(ok1738) III* | many | 0 | Receptor Tyrosine Kinase |
| MT4434 | *ced-5(n1812) IV* | few | 15* | DOCK |
| NW1549 | *efn-2(ev658) IV; efn-3(ev696) X* | few | 0 (n=1) | Ephrin Ligand |
| CB3257 | *ced-2(e1752) IV* | few | 0 | SH2/SH3 adapter |
| CX51 | *dyn-1(ky51) X* | few | 0 | Dynactin |
| CZ414 | *vab-1(e699) II* | few | 0 | Ephrin Receptor |
| MT5267 | *soc-1(n1789) V* | few | 0 | Pleckstrin homology |
| RB1267 | *ensh-1 (ok1349) X* | few | 0 | Fibrogenin like |
| RB1591 | *ddr-1(ok1956) X* | few | 0 | Tyr kinase |
| RB1751 | *rga-5(ok2241) IV* | few | 0 | RhoGAP |
| RB689 | *pak-1(ok488) X* | few | 0 | Ser/Thr kinase |
| RB759 | *akt-1(ok525) V* | few | 0 | Ser/Thr kinase |
| VC674 | *sorb-1(gk304) IV* | few | 0 | SH3 adapter |
| ZD500 | *hecw-1(ok1347) III* | few | 0 | E3 Ubiquitin Ligase |
| RB776 | *kin-32(ok166) I* | few | 0 | Tyr kinase |
| VC664 | *ras-1(ok977) II* | none | 18 | GTPase |
| NW1550 | *efn-2(ev658) IV; him-5(e1490) V* | none | nd |  |
| RB1100 | *ver-4(ok1079) X* | none (n=1) | 0 |  |
| BA1090 | *cav-2(hc191) V* | none | 0 |  |
| JT6130 | *hsp-90(p673) V* | none | 0 |  |
| MJ563 | *tpa-1(k530) IV* | none | 0 |  |
| MT1079 | *egl-15(n484) X* | none | 0 |  |
| NG324 | *wsp-1(gm324) IV* | none | 0 |  |
| PS189 | *let-23(sa62) II* | none | 0 |  |
| PS2728 | *sli-1(sy143) X* | none | 0 |  |
| RB1679 | *cav-1(ok2089) IV* | none | 0 |  |
| RB2027 | *src-1(ok2685) I* | none | 0 |  |
| RB2088 | *F11E6.8(ok2754) IV* | none | 0 |  |
| RB2625 | *F40F12.7(ok3684) III* | none | 0 |  |
| RB783 | *scd-2(ok565) V* | none | 0 |  |
| RB788 | *F11D5.3(ok574) X* | none | 0 |  |
| RB796 | *sta-1(ok587) IV* | none | 0 |  |
| VC126 | *rac-2(ok326) IV* | none | 0 |  |
| VC127 | *pkc-2(ok328) X* | none | 0 |  |
| VC1587 | *C16C2.4 ; ocrl-1(gk752) I* | none | 0 |  |
| VC204 | *akt-2(ok393) X* | none | 0 |  |
| VC2167 | *gck-2(ok2867) V* | none | 0 |  |
| VC2570 | *Y92H12A.2 (ok3321) I* | none | 0 |  |
| VC259 | *pak-2(ok332) V* | none | 0 |  |
| XR1 | *abl-1(ok171) X* | none | 0 |  |
| VC2149 | *sqst-1(ok2869) IV* | none | ~50% Very Dpy | (Eri?), Sequestosome |
| VC1462 | *max-2(ok1904) II* | none | Very Dpy | (Eri?), PAK Kinase |
| RB552 | *aap-1(ok282) I* | none | More Dpy | (Eri?), PI Kinase Regulator |
| RB1566 | *F09A5.2(ok1900) X* | none | More Dpy | (Eri?), Receptor Tyrosine Kinase |
| MT5013 | *ced-10(n1993) IV* | none | More Dpy | (Eri?), RAC GTPase |

* Partial Dpy's,
